# Patient satisfaction in an acute medicine department in Morocco

**DOI:** 10.1186/1472-6963-10-149

**Published:** 2010-06-02

**Authors:** Ghizlane Soufi, Jihane Belayachi, Salma Himmich, Samir Ahid, Mehdi Soufi, Aicha Zekraoui, Redouane Abouqal

**Affiliations:** 1 Medical emergency department, Ibn Sina University Hospital, 10000, Rabat, Morocco; 2 Laboratory of Biostatistics, Clincial and Epidemiological Research, Faculté de Médecine et de Pharmacie - Université Mohamed V, 10000, Rabat, Morocco

## Abstract

**Background:**

Patients' satisfaction is an important indicator for quality of care. Measuring healthcare quality and improving patient satisfaction have become increasingly prevalent, especially among healthcare providers and purchasers of healthcare. This is mainly due to the fact that consumers are becoming increasingly more knowledgeable about healthcare. No studies of inpatients' satisfaction with hospital care have been conducted in Morocco. The first objective of the present study was to confirm the reliability and validity of the Arabic version of the EQS-H (Echelle de Qualité des Soins en Hospitalisation). The second objective was to evaluate patient satisfaction in an acute medicine department in Morocco by using the EQS-H questionnaire; and also to assess the influence of certain demographics, socioeconomics, and health characteristics in patient satisfaction.

**Methods:**

it was a patient survey conducted in an acute medicine department of a Moroccan University Hospital. We surveyed their socio demographic status, and health characteristics at admission. We performed structured face to face interviews with patients who were discharged from hospital. The core of the EQS-H questionnaire was translated to Arabic, adapted to the present setting, and then used to measure patient satisfaction with quality of care. The internal consistency of the EQS-H scale was assessed by Chronbach's coefficient alpha. Validity was assessed by factor analysis. Factors influencing inpatients' satisfaction were identified using multiple linear regression.

**Results:**

The Arabic version of EQS-H demonstrated an excellent internal consistency for the two dimensions studied (0.889 for 'quality of medical information' (MI) and 0.906 for 'Relationship with staff and daily routine' (RS)). The principal component analysis confirmed the bidimensional structure of the questionnaire and explained 60% of the total variance. In the univariate analysis, urban residence, higher income, better perceived health status compared to admission, better perceived health status compared to people of the same age, and satisfaction with life in general were related to MI dimension; Otherwise, mal gender, urban residence, higher income, staying in double room, better perceived health status compared to admission, and satisfaction with life in general were related to RS dimension. The multiple linear regression showed that four independent variables were associated with higher satisfaction in MI: More than 2 prior hospitalizations, a longer length of stay (10-14 days) (*P *= 0.002), staying in double room (*P *= 0.022), and better perceived health status compared to admission (*P *= 0.036). Three independent variables were associated with higher satisfaction in RS: a longer length of stay (10-14 days) (*P *= 0.017), better perceived health status compared to admission day (*P *= 0.013), and satisfaction with life in general (*P *= 0.006).

**Conclusions:**

Our current data assessing patient satisfaction with acute health care by the Arabic version of the EQS-H showed that the satisfaction rate was average on MI dimension; and good on RS dimension of the questionnaire. The majority of participants were satisfied with the overall care. Demographic, socioeconomic, and health characteristics may influence in-patients satisfaction in Morocco, a low/middle income country. An appreciation and understanding of these factors is essential to develop socio culturally appropriate interventions in order to improve satisfaction of patients.

## Background

Respect for patients' needs and wishes is central to any humane health care system [1]. Quality of health services was traditionally based on professional practice standards, however over the last decade, patients' perception about healthcare has been predominantly accepted as an important indicator for measuring quality of health care and a critical component of performance improvement and clinical effectiveness [2]. Measuring healthcare quality and improving patients' satisfaction have become increasingly prevalent, especially among healthcare providers and purchasers of healthcare, because consumers becomes more knowledgeable about healthcare [3]. Indeed, patient satisfaction is widely considered as an integral part of quality of care [1]. Pascoe has defined it as a health care recipient's reaction to salient aspects of his or her experience of a service. In his formulation, satisfaction is assumed to consist of a cognitive evaluation and an emotional reaction to the structure, process and outcome of services [4].

Since the 1990s, measuring patient satisfaction has come to be regarded as a method of choice for obtaining patients' views about their care and has been adopted widely as an outcome indicator of quality of care [5,6]. Most researchers agree that patient satisfaction is a multidimensional concept; however, no consensus exists regarding which dimensions of care should be evaluated to measure patient satisfaction [7,8].

Several approaches have been used to identify the factors contributing to satisfaction with healthcare [9]. A distinction is made between those based on expectations, those focusing on health service attributes, those emanating from economic theory, and those that are holistic in nature [9]. Approaches based on health service attributes attempts to clarify the concept of satisfaction; they also focused on consumers' evaluations of health service attributes. These methods use reviews of the available literature or primary research techniques to produce lists of critical features that affect satisfaction with healthcare. These features are often incorporated into factor or principal components analysis to validate definable dimensions to the care process. The classifications produced may subsequently form the basis for the development of instruments to measure satisfaction [10-12].

In line with previous studies and the literature [13,14], demographics, socioeconomics, and health characteristics in relation to patient, and EQS-H scale were explored. The EQS-H questionnaire is a self-report instrument comprising 16 items, it covers two very important domains of patient satisfaction: "Quality of medical information" (MI) and "Relationship with staff and daily routine" (RS). These two domains (MI and RS) are related to interpersonal aspects of care, which both predictors of patient opinion on care [15]. The EQS-H is an in-patient global satisfaction questionnaire, that should be applicable to most patients admitted to hospital units, whatever their autonomy [16]. The dimensions explored by the EQS-H are not limited to the French healthcare system. Further scale validation in other countries and cultures is required, since it would facilitate cross-cultural studies of health care service quality [16]. Because of the lack of EQS-H instruments in Arabic, this paper presents and discusses the EQS-H adapted into Arabic in terms of applicability and subject acceptance, psychometric performance and validity; as well as the cross-sectional relationship with a selected list of demographic, socioeconomic, and health characteristics.

Morocco has a total population of 31,285,174, gross national income per capita is $ 3.860. The health budget corresponds to 1.1 percent of gross domestic product and 5.5 percent of the central government budget. Morocco has inadequate numbers of physicians (0.5 per 1,000 people) and hospital beds (1.0 per 1,000 people) and poor access to water (82% of the population) and sanitation (75% of the population). The health care system includes 122 hospitals, 2.400 health centers, and 4 university clinics, but they are poorly maintained with inadequate capacity to meet the demand for medical care. Only 24.000 beds are available for 6 million patients seeking care each year, including 3 million emergency cases [17]. Morocco has two major health sectors, public and private, said to be complementary rather than competitive. Patients may choose whether to attend primary or secondary, public or private care. The majority of Moroccans in employment pay for health insurance, which covers most, but not all, of health expenses within the public and private sector. What patient satisfaction face to these conditions and what influence to these conditions in patient satisfaction. To our knowledge, no study concerning the measurement of patient satisfaction has been carried in Morocco. The first objective of the present study was to confirm the reliability and validity of the Arabic version of the EQS-H. The second objective was to evaluate patient satisfaction in an acute medicine department in Morocco using the EQS-H questionnaire and to assess the influence of certain demographics, socioeconomics, and health characteristics in patient satisfaction.

## Methods

### Study Design and Setting

This was a survey of patients conducted in an acute medicine department of Rabat University Hospital between September 2008 and January 2009. The unit admits approximately 950 patients annually with an average age of 40 years. Patients are admitted either from the emergency unit or as non-emergency cases (planned). The service comprises 7 double rooms and 4 common rooms (6 beds per room) and admits patients exhibiting different medical illnesses. Rabat University Hospital is a referral for habitants in western-north Morocco. The study was approved by the local ethics committee and informed consent was obtained from all patients.

### Inclusion and Exclusion Criteria

The study was conducted among patients aged more than 18 years who remained in the hospital longer than 48 hours before they were discharged to their home. Patients with serious physical or mental pathologies, such as terminal disease and psychosis that could make the comprehension and completion of the questionnaire difficult, were also excluded.

### Data Collection

Demographic characteristics included age, gender, marital status (unmarried/married), residence (urban/rural), and distance from patient's home to hospital (≤50 km, >50 km). Socioeconomic characteristics included education level (no education, primary, secondary and more), monthly income (none, less than 180 euro, more than 180 euro), health insurance status (yes, no), and prior hospitalizations in the hospital studied (none, less than 2, more than 2). Health characteristics measured at admission included type of admission (emergency or scheduled), admission room (double/common). All this data were collected on admission. The day before discharge, patients were approached by independent, trained and research assistants. They explained the purpose of the study when patients agree to answer; they invited them to take part and interviewed them face-to-face inside the meetings, and courses room. Most intensive contact so can probe more captures people who are unable to use selfcompletion questionnaire [18]. Studies which used a face to face approach to either subject recruitment (mean response rate: 76.7%) or data collection (mean response rate: 76.9%) were associated with significantly higher response rates than those in which subjects were recruited by mail (mean response rate: 66.5%) or data were collected by mail (mean response rate: 67%) [19]. The timing of questionnaire administration plays an important role. This period should not be too long, so that the answers are specific to the hospitalization [20]. We administered the questionnaire to patients on the day of discharge, in order to obtain a higher response rate. Interviewing patients during their consultations visit at 2 weeks after discharge may result in a lower participation rate; besides, some patients may not return. Crow et al [18], in analyzing 4 studies assessing the administration time of the questionnaire among hospitalized patients have not shown conclusive results. After completion of the questionnaire, other variables were collected. Quality of life characteristics included perceived health status compared to admission (same, little better, much better), perceived health status compared to people of the same age (better, same, worse), and satisfaction with life rated with a scale of 1 to 10. An open-ended question was asked (In your opinion, what would be the priorities for improvement in this department). Length of stay in service (Less than 6 days, 7 to 9 days, 10 to 14 days, More than 14 days) has also been assessed.

### Instrument

The EQS-H is one of the well-known scales usually used to assess inpatient satisfaction with quality of medical and nursing care within hospitals. The EQS-H questionnaire is a self-report instrument comprising 16 items, covering two very important domains of patient satisfaction, "Quality of medical information" (MI) (8 items) and "Relationship with staff and daily routine" (RS). It consists on 16 items; each item is rated on a five-point scale ranging from 1 to 5 (poor, moderate, good, very good and excellent), the final satisfaction score is calculated as the sum of all 16 items scores [16]. This allows calculating 3 scores: a score for each dimension and an overall score. The score of each dimension varies from 8 to 40 and the final score to 80 (with 16 being the minimum and 80 being the highest level of satisfaction equal to 100%).

This format is considered to be the best way to avoid a ceiling effect, often highlighted in satisfaction questionnaires [18,21]. There is consistent evidence across settings that the most important health service factor affecting satisfaction is the patient practitioner relationship, including their primary role in information provision [18,22]. The EQS-H is an in-patient global satisfaction questionnaire, which should be applicable to most patients admitted to hospital units, whatever their autonomy [16]. It explores patient satisfaction toward medical and nursing care received during hospitalization in a short-stay medical or surgery department. We chose the "EQS-H" new version [16] for several reasons. First, it is a short and simple questionnaire that assesses two major components of satisfaction with only 16 questions. Indeed, there is consistent evidence that the most important dimensions of satisfaction are patient-practitioner relationship and interpersonal aspects of care [14,20,23]. Because no Arabic version of the EQS-H was available in the beginning of the study, translation procedures followed by a transcultural adaptation were undertaken following international guidelines [24]. The following steps were used; In the first phase, the EQS-H was translated by three bilingual individuals. All three were native Arabic speakers with excellent proficiency in French. Two individuals were graduate students at the French University. The third translator was a physician. Once the three translations were completed, discrepancies between them were resolved by a committee consisting of the translators and three further individuals not involved in the translation process (a sociologist and two epidemiologists). The committee created one unified translation of the EQS-H. Because of the difficulties related to Arabic grammar and to the style of Arabic writing, two other Arabic linguistics experts also reviewed the translated version. Then, the Arabic version of the EQS-H was backtranslated by a native French speaker living in Morocco, who was unaware of the original French language document. Once the backtranslation completed the committee reconvened to review and resolve the discrepancies between backtranslation and the original document. Finally, a pretest was conducted with a group of lay native Arabic speakers (30 subjects).. For each item the group was asked to explain how it was understood. Overall, few problems were noted. Discrepancies were resolved by group consensus. The committee overseeing the translation process reviewed the final translation. Globally, the adaptation did not cause any particular problems. (See in Additional file 1 Arabic version of the EQS-H questionnaire).

### Statistical Analysis

Data are presented as mean ± standard deviation or frequency values expressed as a percentage. The internal consistency of the EQS-H items was assessed using Chronbach's coefficient alpha; a high alpha coefficient (≥0.70) suggests that the items within a scale measure the same construct and support the construct validity [25,26].

The factor structure of the questionnaire was extracted by the performing both exploratory factor analysis (EFA) and confirmatory factor analysis (CFA). Exploratory factor analysis was performed using the principal component analysis with varimax rotation. Confirmatory factor analysis was performed while a two factor model (MI and RS) was specified for the analysis [27]. To assess the fit of model to our data adequately, we computed Goodness-of-Fit Index (GFI), Comparative Fit Index (CFI), and Root Mean Square of Approximation (RMSEA). The recommended cuts off values for acceptable values are ≥0.90 for GFI and CFI. Cut off value <0.05 indicate a close fit and values below 0.11 are an acceptable fit for RMSEA [27,28].

Concerning univariate analysis, statistical difference between groups were evaluated by the t-student test for comparison between 2 groups, and one-way analysis of variance (ANOVA) for more than 2 groups. The Pearson correlation coefficient (*r*) was calculated to describe the relationships between satisfaction with life and two domains of patient satisfaction: MI and RS. Variables with a *P *≤ 0.25 by univariate analysis were selected for inclusion in a multivariate analysis. Multivariate analysis was performed using multiple linear regression analysis. ί-regression coefficients and their significance from multiple linear regression analysis were reported. A two-tailed *P *< 0.05 was considered statistically significant.

All statistical analyses were carried out using SPSS for Windows 13.0 (SPSS) (SPSS, Inc., Chicago, IL, USA) and AMOS version 4.0 [29].

## Results

### Characteristics of subjects

Three hundred patients were discharged during the study period. Thirty stayed less than 48 hours before they were discharged to their home. Six patients had mental disability, one patient had serious physical disease, 49 patients rejected the participation in the study naming various reasons such as lack of time or simply unwillingness to participate in the study. Face to face interviews were carried out in 214 cases, of which 46.3% were men and 53.7% were women, the mean of age was 45.4 ± 19.6 years. The demographic, socio economic and health characteristics are summarized in table 1.

**Table 1:** Univariate analysis of predictors of satisfaction related to demographics, socioeconomics, and health characteristics.

Characteristics	N	%	MI	RS
**Age(years)**^**a**^				
<25	35	16.4	19.6 ± 7.2	21 ± 7.4
25-39	58	27.1	19.7 ± 5.8	21.8 ± 6.1
40-59	55	25.7	19.8 ± 6.2	24 ± 7.1
≥60	66	30.8	20.1 ± 6.1	22.6 ± 7
*P *value			0.98	0.98
**Gender**				
Male	99	46.3	20.4 ± 6.3	22.3 ±7.4
Women	115	53.7	19.4 ± 6.1	21.6 ± 6.8
*P *value			0.923	0.032
**Marital status**				
Unmarried	66	30.8	19.8 ± 6.6	22.3 ± 7.4
Married	148	69.2	19.8 ± 6	22.6 ± 6.7
*P *value			0.923	0.6
**Residence**				
Urban	134	62.6	20.9 ± 6.5	23.5 ± 7.2
Rural	80	37.4	18 ± 5.1	20.9 ± 6
*P *value			0.001	0.008
**Distance patient's home hospital**				
≤50 km	149	69.6	19.9 ± 6.1	22.7 ± 6.8
>50 km	65	30.4	19.4 ± 6.4	22.2 ± 7.1
*P *value			0.329	0.230
**Education level**				
No education	111	51.9	22 ± 6.4	18.8 ± 5.8
Primary	46	21.5	21 ± 7	25 ± 7.5
Secondary and more	57	26.6	20.7 ± 5.9	21.5 ± 6.9
*P *value			0.48	0.18
**Monthly income**				
None	137	64	19.1 ± 5.6	21.2 ± 6.1
Less than 180 euro	50	23.4	19.9 ± 7.4	24.5 ± 7.8
More than 180 euro *P *value	27	12.6	23 ± 6 0.013	25.3 ± 7.4 0.001
**Health insurance status**				
Yes	43	20.1	21.3 ± 6.4	23.6 ± 7.3
No	171	79.9	19.4 ± 6.1	22.2 ± 6.7
*P *value			0.08	0.22
**Prior hospitalization**				
None	112	52.3	19.8 ± 6.3	22.7 ± 6.6
Less than 2	73	34.1	18.9 ± 5.8	21.6 ± 7.4
More than 2	29	13.6	21.8 ± 6.5	24 ± 6.7
*P *value			0.107	0.25
**Type of admission**				
Emergency	149	69.6	19.9 ± 6.3	22.7 ± 6.8
Scheduled	65	30.4	19.4 ± 6	22.1 ± 7
*P *value			0.58	0.55
**Admission room**				
Double	60	72	21.6 ± 6	24.9 ± 6.2
Common	154	28	19.9 ± 6.3	22.1 ± 7
*P *value			0.09	0.001
**Length of stay (days)**^**a**^				
Less than 6 days	65	30.4	18.7 ± 6	21.6 ± 7.2
7 to 9	45	21	20.3 ± 6.3	23.3 ± 5.8
10 to 14	53	24.8	21.4 ± 7	23.9 ± 7.6
More than 14	51	23.8	19.4 ± 5	21 ± 5.8
*P *value			0.11	0.09
**Perceived health status compared to admission**				
Same Little better	15 92	7 43	15.8 ± 5.5 18 ± 5.6	19.4 ± 4.4 20.9 ± 6.5
Much better	107	50	21.9 ± 6.1	24.3 ± 7
*P *value			<0.001	<0.001
**Perceived health status compared to people of the same age**				
Worse	89	41.6	18 ± 5.4	21.4 ± 6.1
Same	100	46.7	20.6 ± 5.9	23 ± 6.9
Better	25	11.7	22.7 ± 8.2	24.6 ± 8.5
*P *value			0.001	0.075
**Satisfaction with life**				
r			0.172	0.274
*P *value			0.012	<0.001

**Total**	214	100%		

MI subscale: quality of medical information. RS subscale: relationships with staff and daily routine. N:Number. %: Percentage. r: Pearson correlation coefficient. ^a ^stratified by interquartile range. Student *t *test, or analysis of variance (ANOVA).

### Psychometric properties of EQS-H scale

#### Reliability

The internal consistency levels (Chronbach's alpha coefficients) for the two factors were 0.889 for MI and 0.906 for RS. The Chronbach alpha coefficient for the overall satisfaction scale was 0.919.

#### Factor structure

Principal component analysis with varimax rotation loaded two factors. The results are summarized in table 2. Eigen values for the two factors that explained most of the variance observed was 4.52 for RS dimension and 1.37 for MI dimension. The two factor structure (MI and RS) jointly accounted for 59.81% of the variance (table 2). Finally, the confirmatory factor analysis of the two factor model, that is MI and RS, was specified and tested. The results provided a good fit to the data lending support to the original hypothesized structure of the questionnaire with GFI = 0.96, RMSEA = 0.090, and CFI = 0.93.

**Table 2:** Results of factor analysis of EQS-H questionnaire

	Factor loading	
	**Factor 1**	**Factor 2**

**Dimensions/items**		

**I received clear information about:**		
My symptoms		0.612
The purpose of the tests		0.775
The results of the tests		0.788
Purpose of the treatments		0.687
The possible side-effects of these treatments		0.712
Warning signs to look for		0.776
When to resume activities after discharge		0.728
Medical follow-up		0.634
**The hospital staff and the ward:**		
I could identify the doctor in charge of me	0.641	
There was enough privacy during medical care	0.750	
I received enough help in my daily routine	0.597	
Assistance for pain relief	0.740	
The promptness of nurses in coming when called	0.847	
The organization of the ward	0.788	
The atmosphere of the ward	0.804	
The readiness of nurses to spend time with me	0.781	

Percentage of explained variance	31.36	28.45
Mean score ± Standard deviation	22.5 ± 6.9	19.8 ± 6.2
Cronbach alpha	0.906	0.889

#### Descriptive statistics of the subscales

The questionnaire had been generally well accepted by all patients. The mean duration of administration of EQS-H Arabic version was 5.8 ± 3.4 minutes. It has been correctly completed by 97% of patients with no missing or confusing item. The mean value of the total score of EQS-H was 42.5 ± 13.1 [range; 19-79]. The highest mean value (3.6) was observed for the item (I could identify the doctor in charge of me) which is part of the RS dimension. The mean scores of MI was 19.8 ± 6.2 [range; 8-40], and RS dimensions was 22.5 ± 7 [range; 10-40].

### Overall Satisfaction

The satisfaction rate was average on the MI dimension, and good on the RS dimension of the questionnaire. The majority of participants were satisfied with the overall care (Table 3). 20% of the participants responded to the open-ended question. They complained about the lack of attention, the lack of empathy of nurses especially at night, and the lack of information about the disease. They also complained about the excessive number of patients in the common rooms and about the poor quality of sanitary equipments.

**Table 3:** EQS-H questionnaire and distribution of the replie

	Poor	moderate	good	Very good	excellent
I received clear information about:					
My symptoms	23 (10.7%)	83 (38.8%)	73 (34.1%)	19 (8.9%)	16 (7.5%)
The purpose of the tests	20 (9.3%)	73 (34.1%)	79 (36.9%)	24 (11.2%)	18 (8.4%)
The results of the tests	33 (15.4%)	72 (33.6%)	69 (32.2%)	23 (10.7%)	17 (7.9%)
Purpose of the treatments	22 (10.3%)	81 (37.9%)	72 (33.6%)	25 (11.7%)	14 (6.5%)
The Possible side-effects of these treatments	69 (32.2%)	81 (37.9%)	44 (20.6%)	13 (6.1%)	7 (3.3%)
Warning signs to look for	46 (21.5%)	88 (41.1%)	63 (29.4%)	13 (6.1%)	4 (1.9%)
When to resume activities after discharge	75 (35%)	81 (37.9%)	45 (21%)	7 (3.3%)	6 (2.8%)
Medical follow-up	28 (13.1%)	56 (26.2%)	87 (40.7%)	19 (8.9%)	24 (11.2%)
**The hospital staff and the ward:**					
I could identify the doctor in charge of me	2 (0.9%)	29 (13.6%)	83 (38.8%)	36 (16.8%)	64 (29.9%)
There was enough privacy during medical care	13 (6.1%)	62 (29%)	68 (31.8%)	34 (15.9%)	37 (17.3%)
I received enough help in my daily routine	37 (17.3%)	80 (37.4%)	61 (28.5%)	18 (8.4%)	18 (8.4%)
Assistance for pain relief	22 (10.3%)	83 (38.8%)	66 (30.8%)	25 (11.7%)	18 (8.4%)
The promptness of nurses in coming when called	42 (19.6%)	72 (33.8%)	61 (28.6%)	19 (8.9%)	19 (8.9%)
The organization of the ward	14 (6.5%)	73 (34.1%)	86 (40.2%)	15 (7%)	26 (12.1%)
The atmosphere of the ward	16 (7.5%)	54 (25.2%)	103(48.1)	20 (9.3%)	21 (9.8%)
The readiness of nurses to spend time with me	60 (28%)	70 (32.7%)	54 (25.2%)	14 (6.5%)	16 (7.5%)

EQS-H: Echelle de Qualité des Soins en Hospitalisation. N (%): number of patient (Percentage).

### Determinants of satisfaction

#### Univariate analysis

Univariate analysis of the selected variables showed their relationship with the scores related of our satisfaction questionnaire. Table 1 presents the summary results of univariate analysis.

##### Quality of Medical information

Univariate analysis showed that higher satisfaction in MI dimension was associated to urban residence (*P *= 0.001), higher income (*P *= 0.013), better perceived health status compared to admission (*P *< 0.001), better perceived health status compared to people of the same age (*P *= 0.001), and satisfaction with life (*P *= 0.012).

##### Relationship with staff and daily routine

Univariate analysis showed that higher satisfaction in RS dimension was associated to male gender (*P *= 0.032), urban residence (*P *= 0.008), higher income (>180 euro/month) (*P *= 0.001), staying in double room (*P *= 0.001), better perceived health status compared to admission (P < 0.001), and satisfaction with life in general (*P *< 0.001).

#### Multivariate analysis

We also studied the effect of the previous variables on the satisfaction scores after adjustment by all variables in a multivariate model. Table 4 presents the multiple linear regression results

**Table 4:** Multivariate analysis by relevant variables

	Patient satisfaction questionnaire domains			
	**MI**		**RS**	

**Variables**	**β Coef.**	***P *value**	**β Coef**	***P *value**

**Gender**				
Men	0	-------	0	-------
Women	-0.24	0.8	-0.24	0.8
**Residence**				
Urban	1.584	0.093	-------	-------
Rural	0	-------	-------	-------
**Distance patient's home hospital**				
≤50 km	-------	--------	0.532	0.563
>50 km	-------	-------	0	-------
**Education level**				
No education	------	0	-------	-------
Primary	-------	-------	1.529	0.26
Secondary and more	-------	-------	-1.284	0.365
**Monthly income**				
None	0	-------	0	-------
Less than 180 euro	-0.4	0.699	1.587	0.167
More than 180 euro	1.407	0.240	1.407	0.381
**Health insurance status**				
Yes	0	0	------	------
No	0.416	0.684	-0.437	0.713
**Prior hospitalization**				
None	0	-------	0	-------
Less than 2	-0.326	0.704	0.402	0.672
More than 2	3.15	0.008	1.7	0.193
**Admission room**				
Common	0	-------	0	-------
Double	2.049	0.022	1.459	0.138
**Length of stay (days)**^**a**^				
Less than 6 days	0	-------	0	-------
7 to 9	2.084	0.056	1.861	0.124
10 to 14	3.191	0.002	2.715	0.017
More than 14	1.601	0.117	-0.088	0.938
**Perceived health status compared to admission**				
Same	0	-------	0	-------
Little better	0.803	0.63	1.822	0.326
Much better	3.704	0.036	4.95	0.013
**Perceived health status compared to people of the same age**				
Better	2.528	0.058	0.735	0.619
Worse	0	-------	0	-------
Same	1.04	0.26	0.314	0.764
**Satisfaction with life**	0.208	0.42	0.782	0.006

MI subscale: quality of medical information. RS subscale: relationships with staff and daily routine. β Coef.: Beta coefficient from the lineal general model, after adjustment by all relevant variables. Positive values indicate more satisfaction on that domain for that category; negative values indicate less satisfaction compared with the reference category. ^a ^stratified by interquartile range. 0 indicated reference category

##### Quality of medical information

Linear regression shows that four independent variables were associated with higher satisfaction in MI; more than 2 prior hospitalization (*P *= 0.008), staying in double room (*P *= 0.022), longer length of stay (10-14 days) (*P *= 0.002), and better perceived health status compared to admission (*P *= 0.036).

##### Relationship with staff and daily routine

Three independent variables were associated with higher satisfaction in RS: longer length of stay (10-14 days) (*P *= 0.017), better perceived health status compared to admission day (*P *= 0.013), and satisfaction with life in general (*P *= 0.006)

## Discussion

This study reports the results of the first Moroccan study concerning the patient satisfaction with acute health care using the Arabic version of the EQS-H. The psychometric properties of Arabic version of the "EQS-H" questionnaire were discussed previously and showed excellent reliability; the factor analysis confirmed the bidimensional structure of the questionnaire. The EQS-H is a questionnaire developed and validated in France. English, Spanish and Italian versions of the EQS-H satisfaction scale are already available [16]. This facilitates cross-cultural studies of the quality of health care services. Chronbach's coefficient was excellent: it was respectively 0.92 for MI, 0.93 for RS and 0.95 for the 16 items EQS-H Scale overall. The factor analysis confirmed the bidimensional structure of the questionnaire [16]. Thus, the scales are comparable and have the same reliability. Many studies have evaluated inpatient satisfaction in Western countries [30-34], but little is known about inpatient satisfaction in Arabic countries where socio-cultural values differ from those of foreign countries. However, the overall satisfaction observed in our population is lower than reported in previous studies [30-34]. The satisfaction rate was average on the MI dimension; and good on RS dimension of the questionnaire. The majority of participants were satisfied with the overall care. In a study about overall satisfaction with the health-care system in 21 European Union countries, most respondents rated their health systems positively. In all but five countries, more than half of the respondents reported feeling "very satisfied" or "fairly satisfied" [34]. High percentages of extreme satisfaction were observed in items 9, 10, and 14 (29.9%, 17.3%, and 12.1% respectively) as pertaining to its distribution. These items form part of a relationship with staff and daily routine domain. Elsewhere, an extreme dissatisfaction was noted in items five; six and seven (32.2%, 21.5% and 35% respectively). These items form part of quality of medical information domain. Both of two domains are related to interpersonal aspects of care, which both predictors of patient opinion are on care [15]. Patient satisfaction has been shown to be adversely affected by quality of medical information gathering and giving, most evidence showed that satisfaction correlated positively with medical staff feedback and discussions about care. Theories of human behavior may offer useful means of understanding factors such as motivation and designing strategies to change practice. Affective behavior by the physician was consistently related to satisfaction. Detailed investigations of the nature of this relationship show that respondents appreciate: a personalized approach; affective, trust-generating behavior; and a non controlling physician who listen, imparts information and actively involves the patient. The importance of a patient-centered approach is now widely recognized [35,36].

Seven variables describing demographics, socioeconomics, and health characteristics of patients were significant in at least one satisfaction dimension equation, and variables that appeared in the second dimension were consistent in their directions of influence. The most consistent variables associated with a higher satisfaction were more than 2 prior hospitalization, longer length of stay (10-14 days), staying in double room, and better perceived health status compared to admission in MI dimension. Concerning RS dimension, variables were longer length of stay (10-14 days), better perceived health status compared to admission day, and satisfaction with life in general. As in others studies [1,18], and in contrast to other [32], our study showed that in the univariate analysis men tended to be more satisfied than women particularly in RS dimension. These results might indicate that women expect more than men, or that women have different experiences than men do. In many households, a woman may determine the healthcare provided for their children, spouses, parent, parent-in law and even co-workers based upon her experience or satisfaction level with a provider or facility [1], in our cultural context, this can be tied up in place of "dignity" in the male, the man keeping watching outside his image of strength particularly relationally. As demonstrated by Nguyen Thi et al [1], the residences' origin of the patient influences their satisfaction: townspeople were more satisfied than patients who resided in rural areas for both dimensions. This can be explained by the proximity of the structure and care than urban residents can better discern the different measures of hospitalization. On the other hand, in our economic context, the rural life and especially the preserve of the poorest people, this result also merges with the next. Indeed, in our study, higher income has been associated with greater satisfaction in both dimensions in univariate analysis. This finding in our context may not be surprising given that correlates with financial constraints being the most important patient-related factor. In low/middle countries, financial aspects continue to influence strongly the care of patients, especially those with chronic diseases [37]. As reported in a previous study [1], Patient satisfaction was significantly related to hospitalization in double rooms. This could be explained by the fact that double or single rooms provide patients more quiet; more pleasant and allow greater respect intimacy that would make him happier. Currently, with the evolution of hospital structures, there is a gradual loss of shared rooms in favor of single and double rooms. We found greater satisfaction being expressed for a during of stay averaging 10 to 14 and decreases to less than 9 days and beyond 14 days in quality of medical information domain. This could be due to the fact that the quality of the information provided is not sufficient to satisfy the curiosity of patients, and to explain the short or very long duration of hospitalization. In addition, we found that patients who already had more than 2 previous hospital admissions tended to be more satisfied on RS domain. This is probably due to a decrease of the phobia of hospital, also a better knowledge of the circuit of hospital, understanding the conditions and the workload at hospital and allowing the development of a relational sympathy with staff hospital. As reported in previous studies [38,39], better perceived health status compared to admission has been linked to satisfaction in both dimension. In fact, Perceived improvement in health status at the end of hospitalization was a real relief of suffering and should logically be related to higher satisfaction. One investigator shows patients' ratings of their health status to be better predictors of satisfaction than physician ratings [40]. The available evidence indicates that health status can affect satisfaction, and therefore suggests that accurate interpretations of comparative satisfaction data requires consideration of the illness profile of the population samples involved [40]. Inconsistent with others studies, patient age was not the most frequent predictor of satisfaction of the sociodemographic characteristics considered. Although perceived health status compared to people of the same age was associated in satisfaction in MI domains. Self-perceived health status is not usually considered important in satisfaction studies, general health perception score was a strong predictor here [1]. These findings underscore the importance of measuring and controlling for perceived health status when comparing different patient groups or following a patient group over time. Joining a previous study [16], patient's satisfaction with life in general was more satisfied with the quality of care provided. This is explained by the fact that patient satisfaction in their life has a positive view of their situation allowing them to be as satisfied in relation to care.

## Limitations

This study has at least 5 limitations, some of which are inherent in the survey's methodology. Although our use of the EQS-H to evaluate patients' satisfaction needs in an acute medicine service was prospective, there are some limitations in the study. First, the EQS-H is a self-administered questionnaire. Interview techniques may increase response rates, there is a possibility that interviewers can introduce bias [18]. Respondents may give answers that they feel are socially acceptable rather than stating their true view when in a one to- one situation. They may also be influenced by characteristics and attitudes of the interviewer, particularly in face to face situations [18]. Interviewers themselves can make errors when delivering questions or recording answers. The alternative would have been to exclude low-literacy participants. However, the decision to include these participants was more important and better than the risk of bias because the inclusion of the low-literacy participants was a better representation of Moroccan population. Furthermore, the different data collection methods (self-administration and administration by an investigator) have advantages and disadvantages and no consensus is available concerning the problem of administering questionnaires in low-literacy populations [41]. Second, the EQS-H was administered to patients the day before discharge. One study found a U-shaped curve for patient satisfaction with greater satisfaction being expressed several months after discharge compared to few weeks before [42]. But others did not find that timing had any effect on mean responses. On the other hand, response rates decrease as time passed hospitalization increases, the longer the gap between the use of services and the interview (or the questionnaire), the greater the chance of recall bias [43,44]. Third, the staff was not blinded to the fact that a study was being done on patient satisfaction. Fourth, the study results cannot be generalized to any other setting aside from the study site. Lastly, but more importantly, all the patients were picked from only one service, so the results may provide useful information about only those patient's satisfaction. It is not prudent to generalize the results to the whole of hospital care or Moroccan population.

## Conclusions

The Arabic version of the EQS-H showed comparables properties with the French version. This facilitates cross-cultural research. Our current data assessing patient satisfaction with acute health care by the Arabic version of the EQS-H showed that the satisfaction rate was average on the MI dimension; good on the RS dimension of the questionnaire and the majority of participants were satisfied with the overall care. We have discovered a large number of potential barriers and facilitators that may influence in-patients satisfaction in Morocco, a low/middle income country. Satisfaction is a multi-dimensional concept that is part of complex model. An appreciation and understanding of these factors is essential in order to develop socio-culturally appropriate interventions to improve satisfaction of patients. These data underline cultural specificities and financial constraints of our population. A plan of economic and social development is in action, and to improve the image of the public hospital sector and make it more competitive is among its functions [45]. However, Theories of human behaviour may offer useful means of understanding factors such as motivation and designing strategies to change practice. This suggests, that caregivers should develop structured communication programs considering satisfaction predictors (by the improvement personalized approach, clarify and facilitate the comprehension of medical information in a context where illiteracy is more than half of patients admitted. Whatever the level of development of a country, the importance of a patient-centred approach is now widely recognized.

## Competing interests

The authors declare that they have no competing interests.

## Authors' contributions

SG and BJ contributed equally to the work. HS and MS participated in the acquisition of data. SA and ZA participated in the coordination of data. RA conceived of the study, participated in the design of the study, performed the statistical analysis and interpretation of data, and gave the final approval of the manuscript. All authors read and approved the final manuscript

## Pre-publication history

The pre-publication history for this paper can be accessed here:

http://www.biomedcentral.com/1472-6963/10/149/prepub

## Supplementary Material

Additional file 1 **Arabic version of EQS-H questionnaire: The file presents the access of Arabic version of the questionnaire**. Click here for file
