# Supplementary material for: Patient satisfaction in an acute medicine department in Morocco
Source: BMC Health Serv Res. 2010 Jun 2;10:149. doi: 10.1186/1472-6963-10-149 (PMC2900260; doi:10.1186/1472-6963-10-149)
Supplement: Additional file 1 — Arabic version of EQS-H questionnaire: The file presents the access of Arabic version of the questionnaire. [file 1472-6963-10-149-S1.DOC]

**Arabic version of EQS-H questionnaire**

**1- المعلومات التي أعطيت لكم حول وضعكم الصحي و العلاجات التي ستقدم لكم :**

ما مدى وضوح هذه المعلومات؟

حول أعراضكم المرضية؟ ضعيف متوسط جيد جيد جدا ممتاز

حول أهداف الفحوصات التي أنجزت لكم؟

ضعيف متوسط جيد جيد جدا ممتاز

حول نتائج هذه الفحوصات ؟ ضعيف متوسط جيد جيد جدا ممتاز

حول أهداف العلاج؟ ضعيف متوسط جيد جيد جدا ممتاز

حول الأعراض الجانبية لتلك العلاجات؟

ضعيف متوسط جيد جيد جدا ممتاز

**2- الطاقم الطبي و المصلحة** :

ما مدى رضاكم عن المصلحة و العاملين فيها؟

عن معرفة الطبيب المعالج؟ ضعيف متوسط جيد جيد جدا ممتاز

هل يتم احترام خصوصياتكم ؟ ضعيف متوسط جيد جيد جدا ممتاز

المساعدات المقدمة لكم يوميا (الأكل، الغسل، اللباس) ؟

ضعيف متوسط جيد جيد جدا ممتاز

المساعدات المقدمة إليكم لتخفيف الآلام؟ ضعيف متوسط جيد جيد جدا ممتاز

استجابة الممرضة لندائكم؟ ضعيف متوسط جيد جيد جدا ممتاز

النظام في المصلحة؟ ضعيف متوسط جيد جيد جدا ممتاز

مستوى التفاهم بين العاملين في المصلحة؟ ضعيف متوسط جيد جيد جدا ممتاز

استعداد الممرضة لقضاء الوقت معكم؟ ضعيف متوسط جيد جيد جدا ممتاز

**3- المعلومات المعطاة عن مغادرتكم المستشفى** : ما مدى وضوحها؟

حول الأعراض المتوقعة (التي قد تحصل في المستقبل) ؟

ضعيف متوسط جيد جيد جدا ممتاز

حول الأنشطة التي يمكنكم مزاولتها ( مهنية، رياضية) ؟

ضعيف متوسط جيد جيد جدا ممتاز

حول العناية الطبية؟ ضعيف متوسط جيد جيد جدا ممتاز

**4- رأيكم العام:**

هل يتم اعتبار رأيكم في اتخاد قرارات تهم صحتكم ؟

ضعيف متوسط جيد جيد جدا ممتاز

على العموم، كيف كانت العناية و العلاج ؟

ضعيف متوسط جيد جيد جدا ممتاز

**5- بعض التفاصيل تتعلق بكم وبصحتكم :**

الجنس : ذكر انثى

السن :

هل ثم التخطيط لاستشفائكم ؟ أجل لا -استشفاء عبر المستعجلات-

هل حالتكم الصحية تحسنت عندما كنتم في المستشفى؟

لا نعم قليلا نعم كثيرا

مقارنة مع الأشخاص من نفس سنكم، كيف ترون صحتكم ؟

أقل مثل أفضل

ما مدى رضاكم عن حياتكم بصفة عامة ؟

راض جدا

غير راض

10 9 8 7 6 5 4 3 2 1

في رأيكم، ما هي الأولويات لتحسين ظروف الاستشفاء ؟

……………………………………………………………………………………………………………………………………………………………………………………………………………………………………
